# Atlas of the Postnatal Rat Brain in Stereotaxic Coordinates

**DOI:** 10.3389/fnana.2015.00161

**Published:** 2015-12-23

**Authors:** Roustem Khazipov, Dilyara Zaynutdinova, Elena Ogievetsky, Guzel Valeeva, Olga Mitrukhina, Jean-Bernard Manent, Alfonso Represa

**Affiliations:** ^1^Institut de Neurobiologie de la Méditerranée, Institut National de la Santé et de la Recherche Médicale U-901Marseille, France; ^2^Aix-Marseille University, UMR 901Marseille, France; ^3^Laboratory of Neurobiology, Kazan Federal UniversityKazan, Russia

**Keywords:** rat, neonate, brain, atlas, coordinates, bregma, lamda

The series of atlases of the developing rat brain in stereotaxic coordinates that we propose here^1^^,^^2^ has been conceived as a tool for the exploration of the rat brain *in vivo* during the postnatal period, offering a choice of bregma and lambda as the reference points. We suggest to use this atlas, which provides stereotaxic coordinates, in combination with other histological atlases, where brain structures and boundaries between them were assessed using histochemistry or quantative magnetic resonance techniques, but where stereotaxic coordinates are not available (Ramachandra and Subramanian, 2011; Calabrese et al., 2013; Ashwell and Paxinos, 2015) or provided (at P10, P21, and P39) but not within the bregma-lambda metrics (Sherwood and Timiras, 1970).

The neonatal rat is a powerful model for the study of the early stages of central nervous system development. Comparative developmental studies suggest that the first two postnatal weeks in the rat roughly correspond to the second half of gestation and the early postnatal period in humans, with the electrical brain activity patterns expressed during this period and their developmental trajectories matching the patterns observed in premature human neonates (Clancy et al., 2001, 2007; Khazipov and Luhmann, 2006; Colonnese et al., 2010; Workman et al., 2013) (see also http://www.translatingtime.net/). Recent advances in the understanding of the developing brain functions largely came from the use of techniques to record brain activity from neonatal and adolescent rats *in vivo*, notably in head restrained animals (Leinekugel et al., 2002; Khazipov et al., 2004; Minlebaev et al., 2011; Tiriac et al., 2012; Yang et al., 2013). However, the rat brain grows rapidly and non-proportionally during the postnatal period and brain structures change their position in relation to the conventional skull marks such as bregma and lambda, making difficult to perform targeted recordings, stimulations, lesions, or local drug injections in precise anatomical locations. Hence the importance of a developmental atlas of the rat brain in stereotaxic coordinates.

Several examples of histology atlases series of the developing rat brain have been published (Table 1). The Atlas by Sherwood and Timiras “A Stereotaxic Atlas of the Developing Rat Brain” atlas includes three postnatal time points: postnatal days P10, P21, and P39 (Sherwood and Timiras, 1970). Ashwell and Paxinos' “Atlas of the Developing Rat Nervous System” provides photographs and accompanying diagrams of coronal and sagittal sections of rats aged E12, E13, E14, E16, E17, E19, and P0 (Ashwell and Paxinos, 2015). “Atlas of the Neonatal Rat Brain” by Ramachandra and Subramanian describes rat brains at P1, P7, and P14 (Ramachandra and Subramanian, 2011). More recently, Calabrese, Badea, Watson, and Johnson published their “Quantitative magnetic resonance histology atlas of postnatal rat brain development with regional estimates of growth and variability” based on the study of P0, P2, P4, P8, P12, P18, P24, P40, and P80 rats (Calabrese et al., 2013). Among these atlases, only the atlas by Sherwood and Timiras (1970) provides stereotaxic coordinates and yet for only three postnatal days P10, 21, and P39. Stereotaxic atlases for the early postnatal period are not available, however.

**Table 1:** **Atlases of the developing rat brain**.

**Name of atlas**	**Authors**	**Ages**	**Sectioning**	**Web-link**	**Reference**	**Strength**	**Weaknesses**
A stereotaxic atlas of the developing rat brain	Sherwood and Timiras	P10, P21, P39	Coronal, Saggital	Out of print; Available for purchase only at the bookstores	Sherwood and Timiras, 1970	Good image resolution, structures and boundaries, diagrams, stereotaxic coordinates	No data for <P10 rats
Atlas of the developing rat nervous system (3rd edition)	Ashwell and Paxinos	E12, E13, E14, E16, E17, E19, P0	Coronal, Saggital	Available for purchase at the publisher site: http://store.elsevier.com/product.jsp?isbn=9780123694812&locale=en_AU	Ashwell and Paxinos, 2015	High image resolution, many structures and boundaries, diagrams	No data for >P0 rats, no stereotaxic coordinates
Atlas of the neonatal rat brain	Ramachandra and Subramanian	P1, P7, P14	Coronal, Saggital	Available for purchase at the publisher site: https://www.crcpress.com/Atlas-of-the-Neonatal-Rat-Brain/Ramachandra-Subramanian/9781439840122	Ramachandra and Subramanian, 2011	Nissl-stained plates	No stereotaxic coordinates, only major structures labeled, no diagrams
Quantitative magnetic resonance histology atlas of postnatal rat brain development with regional estimates of growth and variability	Calabrese, Badea, Watson, and Johnson	P0, P2, P4, P8, P12, P18, P24, P40, P80	Multi-dimentional	Open access at: http://www.civm.duhs.duke.edu/ratbraindevatlas/	Calabrese et al., 2013	High magnetic resonance images, multidimen-tionality	No stereotaxic coordinates, only major structures labeled
Atlas of the postnatal rat brain in stereotaxic coordinates	Khazipov, Zaynutdinova, Ogievetsky, Valeeva, Mitrukhina, Manent, and Represa	P0, P1, P2, P3, P4, P5, P6, P7, P8, P9, P10, P14, P21	Coronal	Open access at: www.ial-developmental-neurobiology.com/en/publications/collection-of-atlases-of-the-rat-brain-in-stereotaxic-coordinates	Present report	Stereotaxic coordinates with bregma and lamda reference points	Low image resolution, only major structures labeled, no boundaries, no diagrams

The series of atlases of the developing rat brain in stereotaxic coordinates that we propose here has been conceived as a tool for the exploration of the rat brain *in vivo* during the postnatal period, offering a choice of bregma and lambda as the reference points. Each atlas contains a series of microphotographs of wet, non-stained 200 micron coronal brain sections in oblique light, obtained from postnatal days P0, 1, 2, 3, 4, 5, 6, 7, 8, 9, 10, 14, and 21 rats, with an indication of the main brain structures. Wet non-stained sections were used to avoid deformation, shrinkage and lessening associated with staining and mounting procedures, particularly when manipulating immature tissue. Because we did not stain sections, the images do not provide cellular resolution and not all brain structures and boundaries between them can be easily identified. Therefore, only certain brain structures are labeled without boundaries. Used in combination with the existing high image resolution atlases of the developing rat brain (Sherwood and Timiras, 1970; Ramachandra and Subramanian, 2011; Calabrese et al., 2013; Ashwell and Paxinos, 2015), this atlas may be useful for targeted recordings, stimulations, and drug injections in different brain structures at various postnatal ages. This atlas has been successfully used in several previous studies (Minlebaev et al., 2011; Petit et al., 2014; Tyzio et al., 2014).

## Stereotaxic surgery

All animal-use protocols followed the guidelines of the French National Institute of Health and Medical Research (INSERM, protocol N007.08.01). Wistar rats of both sexes from postnatal days [P] 0, 1, 2, 3, 4, 5, 6, 7, 8, 9, 10, 14, and 21 were used. P0 was the day of birth. Three to five animals per age were used in this study and one animal per age with the best histology quality was selected for the atlas. The surgery was performed under urethane anesthesia (1.5 g/kg). The skull of the animal was cleaned of skin and periosteum. A hemostabe was used to stop bleeding. Two metal bars were fixed to the nasal and occipital bones of the rat's head with dental cement (Grip Cement). During tracing, the head was fixed to the frame of the Kopf stereotaxic apparatus with bars attached similarly to the head fixation used during recordings from neonatal rat pups (Leinekugel et al., 2002; Khazipov et al., 2004; Minlebaev et al., 2011). This approach is different from the conventional method of head fixation with ear and tooth bars used by Sherwood and Timiras (1970), which cannot be used in neonatal animals <P10 because the skull is cartilaginous. Bregma and lambda were adjusted to the same height and laterally aligned using a needle attached to a stereotaxic manipulator to control their vertical and horizontal coordinates. Thus, the horizontal plane of the atlas extends from bregma to lambda. Because of the use of an “artificial skull” for the head fixation, and unlike in other stereotaxic atlases, we do not provide a vertical zero plane.

Holes were drilled in the skull 1–3 mm lateral from the bregma and lambda, through which a needle colored by dipping in an ethanol solution of DiI (1,1′-Dioctadecyl-3,3,3′,3′-tetramethylindocarbocyanine perchlorate, Sigma-Aldrich, St. Louis, USA) was vertically inserted at a depth of 3–5 mm. An additional insertion of the DiI colored needle through the whole brain in a coronal plane was made 3–5 mm lateral from the midline between bregma and lambda. This line was used for the positioning of the brain during coronal slicing.

## Histology

After tracing, rats were intracardially perfused with a solution of 4% paraformaldehyde (pH 7.2–7.4; Antigenfix, Diapath, Italy) and 1–3% glutaraldehyde (Sigma-Aldrich, St. Louis, USA), the brain was removed and left in fixative for 1–6 days depending on the age of the animal. Then brains were rinsed in PBS (prepared from PBS tablets, Gibco, Paisley, UK; pH 7.45, osmolarity 305 mosm) photographed in DiI fluorescent light from above and placed in a block of agar. To ensure that coronal sections were made strictly perpendicular to the horizontal plane, blocks were cut at the bottom parallel to the lateral DiI-labeled trace going through the whole brain. Then the blocks were glued to the metal plate of the vibroslicer (Leica VT1000S) and 200 micron thick coronal sections were cut from the olfactory bulb to the spinal cord. Sections were then placed in a 12 wells plastic dish and kept in PBS. Microphotographs of wet sections were made using OLYMPUS SZX16 microscope equipped with a DP71camera, at 0.7–1.6 zoom in oblique light. During photography, the excess of PBS was removed. No coloration of brain sections was used in this study. Microphotographs were acquired at 150 dpi in .psd format using cellSens Standard 1.6 software.

## Image processing

**Figure 1:**
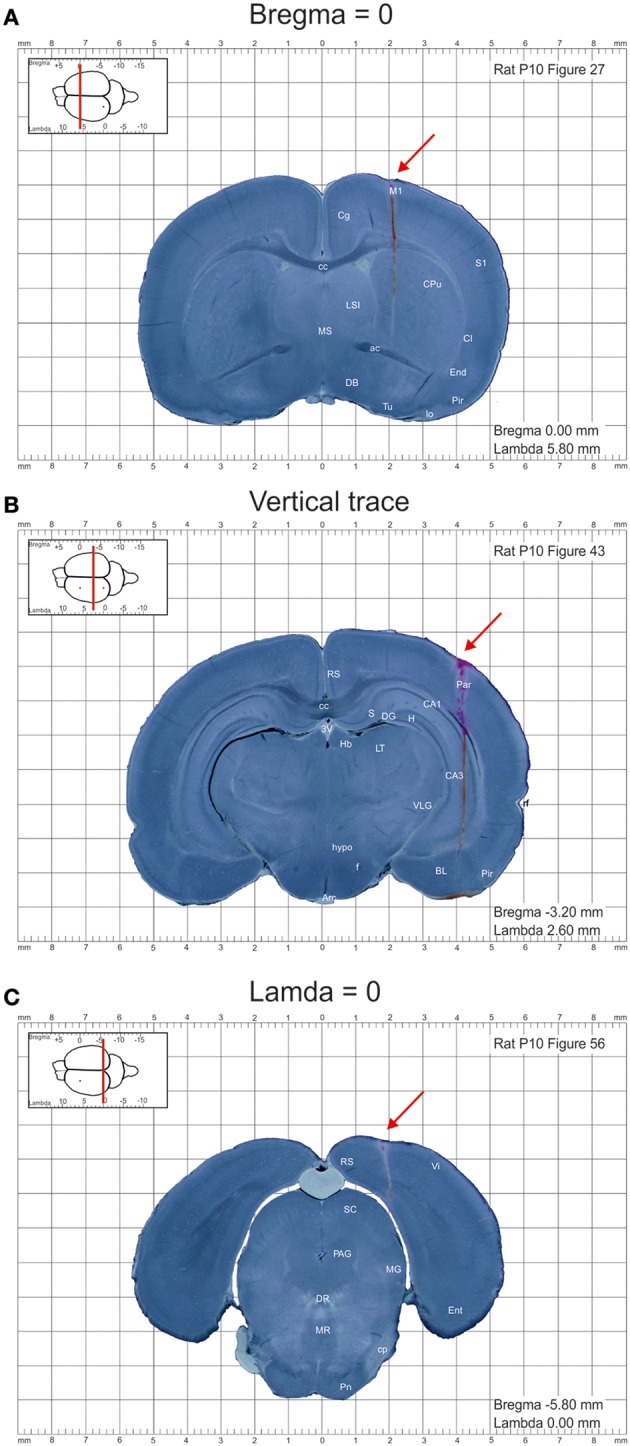
**Example images of the coronal sections from the postnatal day P10 rat brain from the atlas of the developing rat brain in stereotaxic coordinates. (A–C)** Coronal sections at anterioposterior zero distances from the bregma **(A)** and lambda **(C)**, and a section illustrating the trace through the whole brain **(B)**. DiI-labeled penetrations are indicated by red arrows.

Images were further processed using Photoshop CS5.1 software and converted to 150 dpi images in .png format. DiI labeled tracks of the penetrations at bregma and lambda levels were used to calculate the anterioposterior distance of each section from the bregma and lambda, respectively (Figures 1A,C). The entire appearance of the lateral across-brain track in not more than two sections was used to ensure that the sections were cut parpendicular to the horizontal plane (Figure 1B). Otherwise, the brains were discarded from analysis. Images were further assembled into the atlas using InDesigner CS5.5 with an indication of the anterioposterior distance of the section from bregma and lamda and converted to the final.pdf format at 300 dpi resolution.

## Data base description

The collection of atlases prepared from postnatal days P0, 1, 2, 3, 4, 5, 6, 7, 8, 9, 10, 14, and 21 rats is deposited at www.ial-developmental-neurobiology.com/en/publications/collection-of-atlases-of-the-rat-brain-in-stereotaxic-coordinates and http://www.inmed.fr/en/en-atlas-stereotaxique-du-cerveau-de-rat-au-cours-du-developpement-postnatal. Each file contains a series of obtained in oblique light microphotographs of 200 micron wet, non-colored coronal brain sections of a rat at the age indicated in the file name and a list of abbreviations of the main brain structures. For identifying brain structures, sections were systematically compared with images from the developing rat brain atlases (Sherwood and Timiras, 1970; Paxinos et al., 1991; Ramachandra and Subramanian, 2011), adult rat brain atlas (Paxinos and Watson, 2007), and our own stained sections from the age-matching animals used in other studies. Only the brain areas that we could accurately identify were quoted in the study. Atlases are presented in pdf format at 300 dpi resolution. To move along the anterioposterior sagittal plane one can use conventional Adobe view instruments or chose a position by clicking the brain image located at the left top panel.

## Author contributions

RK conceived the project. RK, DZ, GV, and OM performed tracing and prepared brain sections. DZ made photographs of the brain sections. JM and AR marked brain regions. EO processed images and assembled the atlases. RK wrote the paper.

### Conflict of interest statement

The authors declare that the research was conducted in the absence of any commercial or financial relationships that could be construed as a potential conflict of interest.

## Footnotes

^1^www.ial-developmental-neurobiology.com/en/publications/collection-of-atlases-of-the-rat-brain-in-stereotaxic-coordinates;

^2^http://www.inmed.fr/en/en-atlas-stereotaxique-du-cerveau-de-rat-au-cours-du-developpement-postnatal.
